# 2114. Evaluation of Isavuconazole Activity against Non-*fumigatus Aspergillus* and cryptic *Aspergillus* species Causing Invasive Infections Worldwide

**DOI:** 10.1093/ofid/ofad500.1737

**Published:** 2023-11-27

**Authors:** Cecilia G Carvalhaes, Paul Rhomberg, Valerie Kantro, Beth Hatch, Mariana Castanheira

**Affiliations:** JMI Laboratories, North Liberty, IA; JMI Laboratories, North Liberty, IA; JMI Laboratories, North Liberty, IA; JMI Laboratories, North Liberty, IA; JMI Laboratories, North Liberty, IA

## Abstract

**Background:**

Isavuconazole (ISC) is considered first-line therapy for the treatment of invasive aspergillosis (IA). There is limited data on antifungal susceptibility testing of clinically relevant non-*fumigatus* (non-AFM) and cryptic species of *Aspergillus* (cryAsp) causing IA. The activity of ISC and other azoles against non-AFM and cryAsp causing IA worldwide was evaluated.

**Figure:**
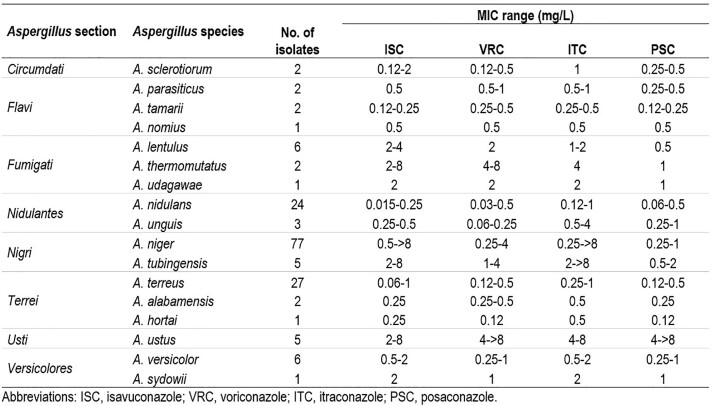

**Methods:**

A total of 390 non-AFM, including 63 cryAsp isolates, were collected (1/patient) in 2017–2021 from 41 medical centers located in Europe (EU; *n*=161; 17 centers), North America (NA; *n*=137; 16 centers), Asia-Pacific (AP; *n*=85; 7 centers), and Latin America (LA, *n*=7; 1 center). Isolates were identified by MALDI-TOF MS and/or ITS/β-tubulin sequencing and tested by CLSI broth microdilution. CLSI epidemiological cut-off values were applied, where available.

**Results:**

ISC showed activity against *A.* sections *Flavi* (*n*=122; MIC_50/90_, 0.5-1 mg/L), *Terrei* (*n*=57; MIC_50/90_, 0.5/0.5 mg/L), *Nidulans* (*n*=34; MIC_50/90_, 0.12/0.25 mg/L), *Versicolores* (*n*=7; MIC_50_, 1 mg/L), and *Circumdati* (*n*=2; MIC range, 0.12–2 mg/L). Similar activity was displayed by other azoles against those *Aspergillus* sections. Most of the isolates from *A.* sections *Fumigati* (*n*=9), *Nigri* (*n*=146), and *Usti* (*n*=12) exhibited elevated MIC values to ISC (MIC_50/90_, 2/8, 2/4, and 2/8 mg/L), VRC (MIC_50/90_, 2/8, 1/2, and 4/8 mg/L), ITC (MIC_50/90_, 2/4, 2/4, and 8/ > 8 mg/L), and PSC (MIC_50/90_, 0.5/1, 0.5/1, and > 8/ > 8 mg/L), respectively. The activity of ISC and other azoles against cryAsp, *A. niger* and *A. terreus*, when identified to the species level, are listed in the Table. ISC was active (MIC values, ≤ 1 mg/L) against *A. parasiticus*, *A. tamarii*, *A. nomius*, *A. nidulans*, *A. unguis*, *A. terreus*, *A. alabamensis*, and *A. hortai,* while ISC MIC values between 2–8 mg/L were observed against cryAsp from *A*. section *Fumigati*. ISC inhibited 96.1% of *A. niger* and 80.0% of *A. tubingensis* at ≤ 4 mg/L, the CLSI wildtype cut-off value for *A. niger*. VRC, ITC, and PSC showed similar activity to ISC against most cryAsp.

**Conclusion:**

ISC exhibited potent *in vitro* activity against non-AFM, including cryAsp. However, the activity of ISC and other azoles vary among and within cryAsp species. Susceptibility testing is critical to guide treatment of cryAsp causing IA.

**Disclosures:**

**Cecilia G. Carvalhaes, MD, PhD**, AbbVie: Grant/Research Support|bioMerieux: Grant/Research Support|Cipla: Grant/Research Support|CorMedix: Grant/Research Support|Melinta: Grant/Research Support|Pfizer: Grant/Research Support **Paul Rhomberg, BS, MT(ASCP)**, bioMerieux: Grant/Research Support|Melinta: Grant/Research Support|Pfizer: Grant/Research Support **Valerie Kantro, BA**, AbbVie: Grant/Research Support|Pfizer: Grant/Research Support|Shionogi: Grant/Research Support **Beth Hatch, BS, MT(ASCP)**, Pfizer: Grant/Research Support **Mariana Castanheira, PhD**, AbbVie: Grant/Research Support|Basilea: Grant/Research Support|bioMerieux: Grant/Research Support|Cipla: Grant/Research Support|CorMedix: Grant/Research Support|Entasis: Grant/Research Support|Melinta: Grant/Research Support|Paratek: Grant/Research Support|Pfizer: Grant/Research Support|Shionogi: Grant/Research Support

